# The interferon-inducible antiviral protein Daxx is not essential for interferon-mediated protection against avian sarcoma virus

**DOI:** 10.1186/1743-422X-11-100

**Published:** 2014-05-28

**Authors:** Kelsey A Haugh, Natalia Shalginskikh, Shoko Nogusa, Anna Marie Skalka, Richard A Katz, Siddharth Balachandran

**Affiliations:** 1 Immune Cell Development and Host Defense Program, Fox Chase Cancer Center, Room 422 Reimann Building, 333 Cottman Ave., 19111 Philadelphia, PA, USA

**Keywords:** Daxx, Interferon, Avian sarcoma virus, Innate immunity

## Abstract

**Background:**

The antiviral protein Daxx acts as a restriction factor of avian sarcoma virus (ASV; *Retroviridae*) in mammalian cells by promoting epigenetic silencing of integrated proviral DNA. Although Daxx is encoded by a type I (α/β) interferon-stimulated gene, the requirement for Daxx in the interferon anti-retroviral response has not been elucidated. In this report, we describe the results of experiments designed to investigate the role of Daxx in the type I interferon-induced anti-ASV response.

**Findings:**

Using an ASV reporter system, we show that type I interferons are potent inhibitors of ASV replication. We demonstrate that, while Daxx is necessary to silence ASV gene expression in the absence of interferons, type I interferons are fully-capable of inducing an antiviral state in the absence of Daxx.

**Conclusions:**

These results provide evidence that Daxx is not essential for the anti-ASV interferon response in mammalian cells, and that interferons deploy multiple, redundant antiviral mechanisms to protect cells from ASV.

## Findings

### Introduction

Avian Sarcoma Virus (ASV) is a prototypic alpharetrovirus (family *Retroviridae*) that can be pseudotyped to transduce mammalian cells and study host antiviral responses. We have previously shown that the cellular scaffolding protein Daxx, originally identified a mediator of death-receptor-triggered apoptosis [1], is also a potent anti-ASV restriction factor [2]. Daxx is recruited to viral DNA by ASV integrase, where it promotes the rapid epigenetic repression of integrated viral DNA via recruitment of gene-repressive histone deacetylases (HDACs) and DNA methyl transferases [2,3]. We identified an essential role for Daxx in controlling ASV replication by demonstrating that genetic ablation or RNA interference-mediated knockdown of Daxx expression resulted in significantly-increased expression of an ASV-encoded reporter gene [2,3].

Type I (predominantly α/β) interferons (IFNs), are a family of cytokines with powerful antiviral and immune-modulatory effects, and are rapidly induced in most cells upon virus infection. Once produced, IFNs activate an antiviral state in the infected cell, as well as in surrounding cells, by Jak/STAT-regulated induction of >1000 IFN-stimulated genes (ISGs) [4,5]. Daxx mRNA and protein expression are induced following exposure to type I IFNs, indicating that *Daxx* is an ISG [2,3].

Here, we demonstrate that type I IFNs are powerful inhibitors of ASV replication in human and avian cells. We show that, although Daxx is upregulated by type I IFNs and essential on its own for silencing ASV gene expression in human cells, it is largely dispensable for establishment of the type I IFN-induced anti-retroviral state. Our results suggest that IFNs are capable of effectively inhibiting ASV even in the absence of Daxx, providing evidence that epigenetic silencing by Daxx is a redundant mechanism of the IFN anti-ASV response in mammalian cells.

### Type I IFNs inhibit ASV replication in mammalian cells

To investigate whether type I IFNs can block the early steps in ASV replication, we treated HeLa cells with either human IFN-α or IFN-β prior to infection with an ASV-GFP reporter virus. This reporter virus is pseudotyped to express the murine leukemia virus (MuLV) amphotropic envelope protein, and is therefore capable of entry into mammalian cells. ASV-GFP contains an intact complement of replicative genes, and is fully-capable of productive infection in its natural avian host cells, but several post-transcriptional blocks in mammalian cells inhibit late events in the virus life-cycle, limiting infection to a single round in these cells [2,3]. ASV-GFP infection of mammalian cells, however, recapitulates key early events of the retroviral life-cycle, including entry, uncoating, reverse-transcription and integration. As diminished GFP expression is a faithful readout of Daxx-dependent silencing, we have previously employed ASV-GFP to identify post-integration silencing of retroviral gene-expression as a Daxx-sensitive step [2,3].

**Figure 1:**
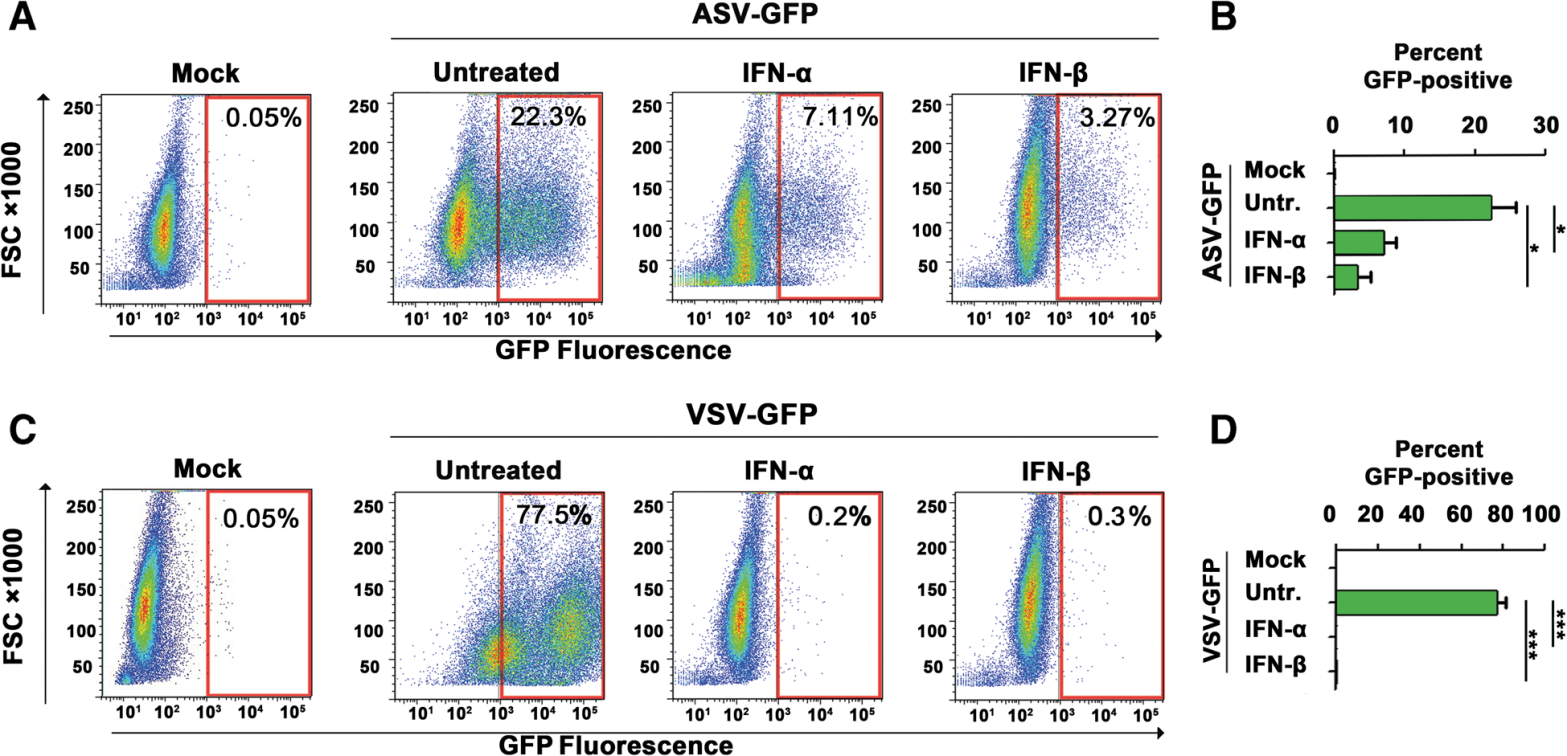
**Type I IFNs inhibit ASV replication. (A)** Fluorescence-activated Cell Sorter (FACS) analysis of ASV-GFP replication (indicated by % GFP-positive cells) in untreated, human IFN-α (1000 U/ml)- or human IFN-β (1000 U/ml)-treated HeLa cells 48 h post-infection from a representative experiment. GFP fluorescence data were collected on an LSR II flow cytometer (Becton Dickinson), and analyzed using FlowJo software. FSC = Forward scatter. **(B)** Quantification of GFP-positive cells from four independent replicates of the experiment described in panel **A**. Error bars represent mean +/- standard deviation. * *p* <0.05. **(C)** VSV-GFP replication (indicated by % GFP-positive cells) in untreated, IFN-α (1000 U/ml)- or IFN-β (1000 U/ml)-treated HeLa cells 24 h post-infection from a representative experiment. **(D)** Quantification of GFP-positive cells from four independent replicates of the experiment described in panel **C**. Error bars represent mean +/- standard deviation. ***p <0.001.

After treating HeLa cells with either IFN-α or IFN-β for 18 h, we infected these cells with ASV-GFP in the presence of DEAE-Dextran (20 μg/mL), as described previously [6], and quantified viral gene expression by measuring GFP fluorescence 48 h post-infection. As the IFN-induced antiviral state is rarely maintained for more than 30 h post-treatment [7], cells were supplemented with IFN 6 h and 24 h post infection. Vesicular stomatitis virus encoding GFP (VSV-GFP) [8] was used as a positive control for IFN activity, as VSV is a well-established IFN-sensitive virus [9,10]. We found that treatment of HeLa cells with either IFN-α or IFN-β efficiently diminished GFP positivity (by ~70% and ~85%, respectively) following ASV infection, demonstrating that type I IFNs are capable of blocking ASV gene expression (Figure 1A,B). As expected, IFN-α and IFN-β inhibited VSV-GFP replication almost completely (from >75% GFP-positive cells in untreated controls to <1% GFP-positive cells after IFN-α/β treatment; Figure 1C,D).

### Type I IFNs Inhibit ASV replication in avian cells

**Figure 2:**
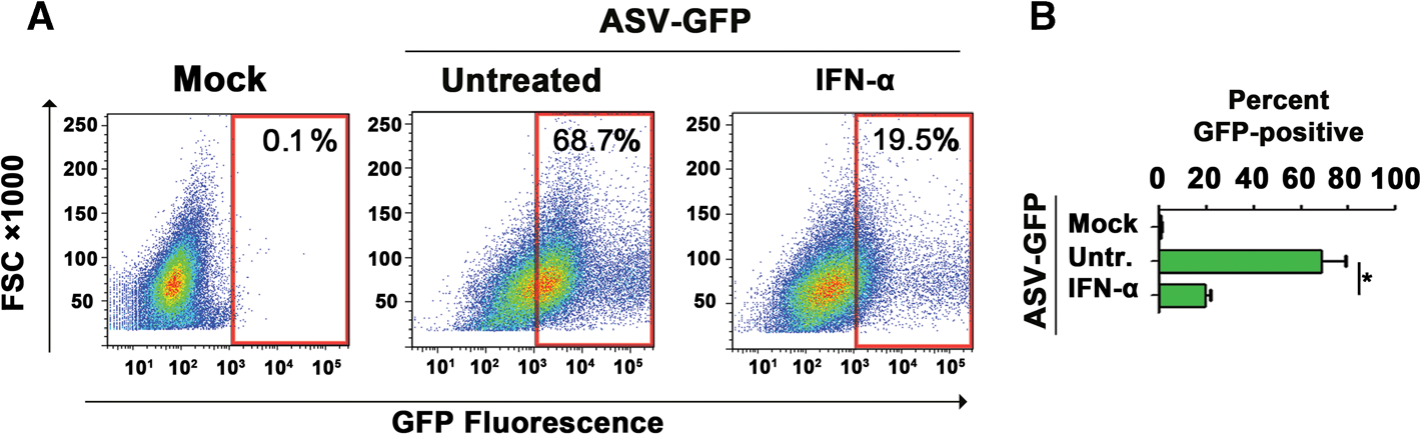
**Chicken IFN-α inhibits ASV replication in DF-1 cells. (A)** FACS analysis of ASV-GFP replication (indicated by % GFP-positive cells) in untreated or chicken IFN-α (1000 U/ml)-treated DF-1 cells 48 h post-infection from a representative experiment. FSC = Forward scatter. **(B)** Quantification of GFP-positive cells from three independent replicates of the experiment described in panel **A**. Error bars represent mean +/- standard deviation. *p <0.05.

To extend this investigation to cells of natural ASV hosts, we performed similar experiments in DF-1 chicken cells. We limited ASV replication to a single round in these cells by using a self-inactivating ASV-based alpharetroviral GFP-transducing vector with diminished LTR transcriptional activity [11]. After treating DF-1 cells with chicken IFN-α for 18 h, we infected these with 5 μL of self-inactivating ASV-GFP in the presence of Polybrene (10 μg/mL) at 37°C for 1 h. To ensure continued maintenance of the antiviral state, we supplemented cells with IFN-α 6 h and 24 h p.i. When we examined these cells by GFP-based flow cytometry 48 h p.i., we observed that treatment with chicken type I IFN diminished proviral reporter gene expression by a significant amount (by ~70%, Figure 2), as observed in mammalian cells (Figure 1A-D). Collectively, these results demonstrate that type I IFNs exert antiviral activity against ASV, and set the stage for experiments designed to determine if Daxx is an essential component of the IFN anti-ASV program.

### Daxx is induced by type I IFNs in mammalian and avian cells

**Figure 3:**
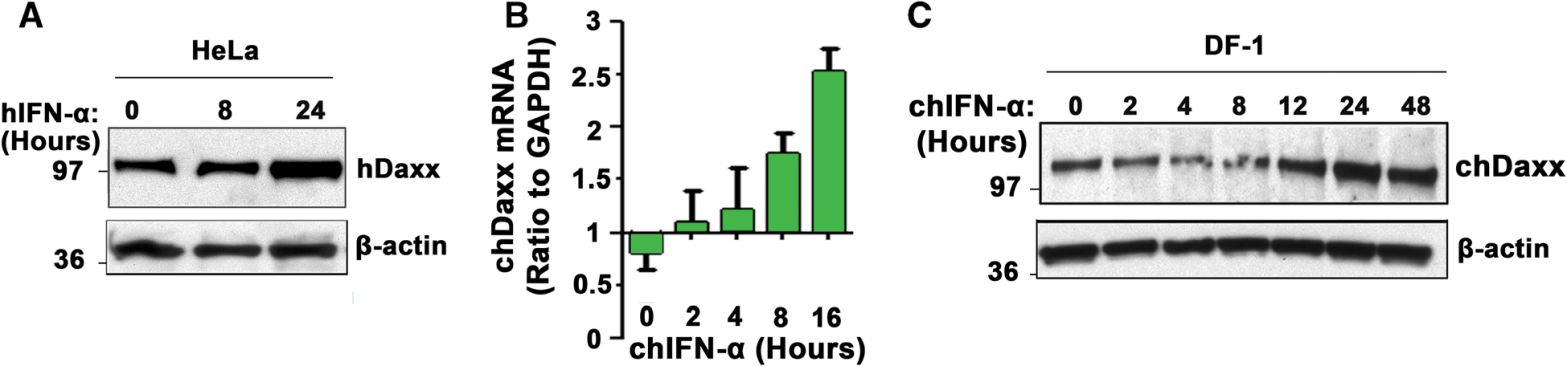
**Daxx is induced by type I IFNs. (A)** Immunoblot analysis of Daxx protein levels (antibody from Sigma) after human (h) IFN-α (1000 U/ml) treatment of HeLa cells for the indicated time points. **(B)** Real-time PCR showing Daxx mRNA levels in DF-1 cells following treatment with chicken (ch) IFN-α for the indicated time points. Values were normalized to GAPDH. **(C)** Immunoblot analysis of Daxx protein levels following chicken (ch) IFN-α treatment of DF-1 cells for the indicated times. β-actin (antibody from Sigma) is shown as a loading control. Molecular-weight markers, in kilodaltons, are shown to the left of each blot.

We previously demonstrated that treatment with IFN-α results in induction of *Daxx* mRNA in HeLa cells [3]. To evaluate Daxx protein levels following IFN treatment, we treated HeLa or DF-1 cells with either human or chicken IFN-α, respectively, and examined whole-cell lysates prepared from these cells at various times post-treatment by immunoblotting. As shown in Figure 3A, IFN treatment increased Daxx protein levels ~3-fold by 24 h in HeLa cells. In DF-1 cells, IFN-α induction of Daxx was confirmed to occur at the mRNA level (~2.5-fold, Figure 3B). A protein band of the approximate size of the putative avian Daxx ortholog was similarly induced by chicken IFN-α (Figure 3C). Thus, Daxx is an IFN-inducible protein in both mammalian and avian cells.

### Daxx is not essential for type I IFN-mediated inhibition of ASV replication in mammalian cells

**Figure 4:**
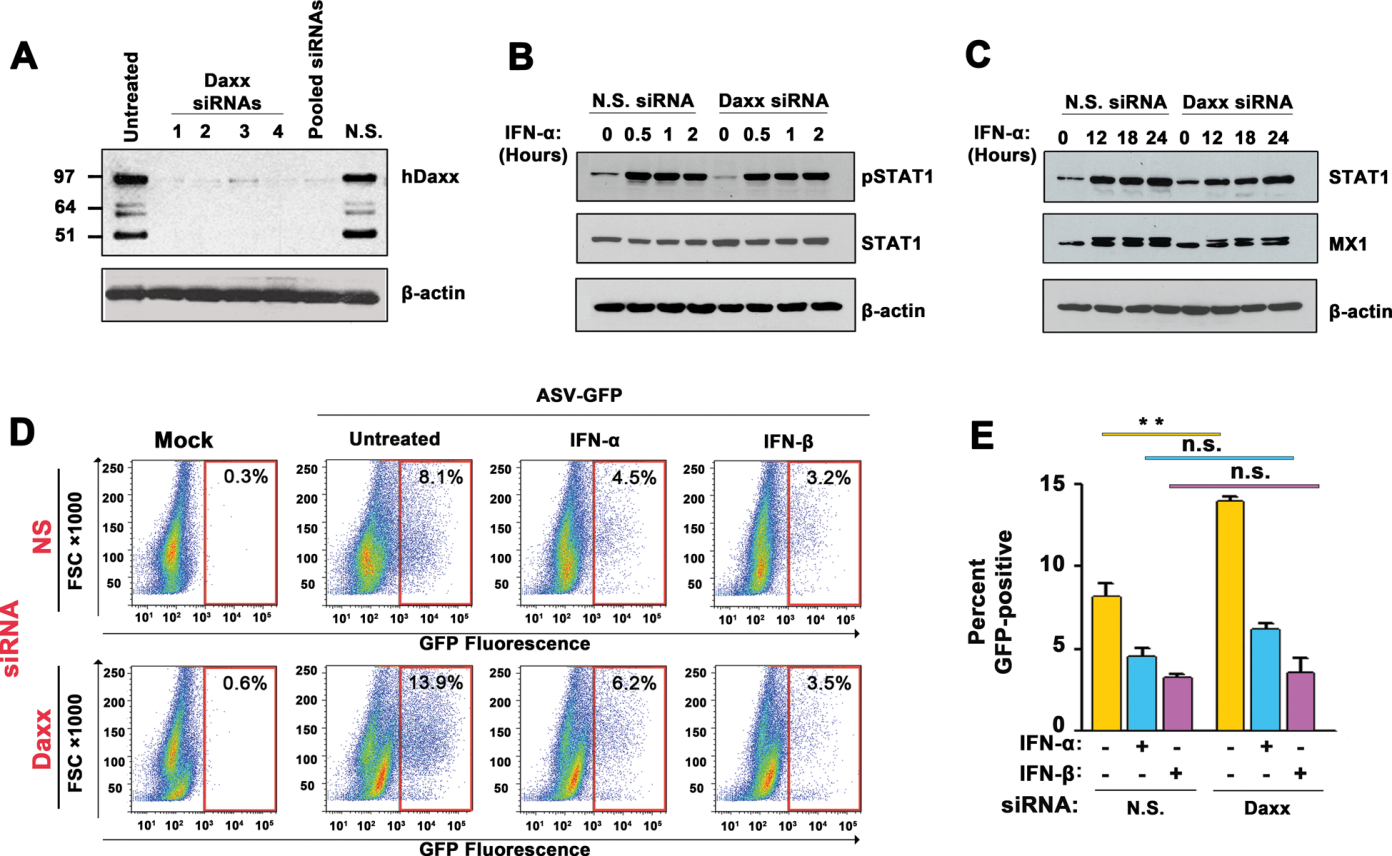
**Daxx is not required for type IFN inhibition of ASV replication in mammalian cells. (A)** Immunoblot showing Daxx protein levels in HeLa cells following transfection of four individual (lanes 2–5) or pooled (lane 6) siRNAs to human Daxx. Lane 1 = untreated control. Molecular weight markers, in kilodaltons, are shown to the left. β-actin was used as a loading control. **(B)** Immunoblot analysis of STAT1 phosphorylation following IFN-α treatment of HeLa cells transfected with nonspecific (N.S.) or pooled Daxx siRNAs. Cells were treated with IFN-α (1000 U/ml) for the indicated time points. β-actin was used as a loading control. **(C)** Immunoblot analysis of STAT1 and MX1 induction following IFN-α treatment of HeLa cells transfected with nonspecific (N.S.) or pooled Daxx siRNAs. **(D)** ASV-GFP replication (indicated by % GFP-positive cells) in untreated, human IFN-α (1000 U/ml)- or human IFN-β (1000 U/ml)-treated HeLa cells 48 h post-infection. Cells were transfected with nonspecific (NS) or Daxx siRNAs for 72 h prior to infection, as indicted to the left. FACS data from are representative experiment are shown. FSC = Forward scatter. **(E)** Quantification of GFP-positive cells from three independent replicates of the experiment described in panel **D**. Error bars represent mean +/- standard deviation. **p <0.01. n.s., not significant.

To directly determine if Daxx contributed non-redundantly to the IFN-induced antiviral state in mammalian cells, we knocked-down *Daxx* expression by RNAi. Individual or pooled transfection of HeLa cells with four distinct siRNAs that target *Daxx* mRNA reduced Daxx protein levels in HeLa cells to nearly-undetectable levels within 72 h of treatment (Figure 4A). By contrast, Daxx levels in cells transfected with control non-silencing siRNAs were comparable to those seen in untreated HeLa cells (Figure 4A). To determine whether Daxx knockdown resulted in an altered ISG induction profile following IFN treatment, we transfected HeLa cells with control or pooled Daxx siRNAs, treated these cells with IFN-α, and analyzed lysates from these cells by immunoblotting. Knockdown of Daxx did not significantly alter the kinetics or magnitude of STAT1 phosphorylation by IFN-α (Figure 4B). Over a longer time course of 24 h, Daxx knockdown did not affect the kinetics of induction of prototypic ISG-encoded proteins STAT1 and MX1, and only modestly affected the magnitude of their induction (Figure 4C). Together, these results indicate that loss of Daxx does not significantly alter IFN-α-induced transcription.To test if Daxx was required for IFN-mediated antiviral activity against ASV, we first transfected HeLa cells with either non-silencing siRNAs, or with a pool of the four siRNAs that target Daxx, and exposed these cells to IFN-α or IFN-β 54 h post-transfection. At 72 h post-siRNA transfection (and 18 h post IFN treatment) when Daxx knockdown was maximal (Figure 4A), cells were infected with ASV-GFP and supplemented with IFN 6 h and 24 h p.i., before analysis by flow cytometry at 48 h p.i. Consistent with previous findings, ASV reporter gene expression was higher in Daxx-knockdown cells compared to those treated with nonspecific siRNA (by about two-fold, Figure 4D,E). However, both IFN-α and IFN-β reduced ASV-GFP expression to approximately the same levels (~5% by IFN-α and ~3% by IFN-β) in cells transfected with siRNA to Daxx as they did in cells carrying non-silencing siRNAs (Figure 4D,E). These results provide evidence that Daxx is not essential for IFN-mediated protection against ASV in mammalian cells.

## Conclusions

The two salient findings of this study are that (1) type I IFNs can potently inhibit ASV replication in mammalian and avian cells, and (2) that although Daxx is IFN-inducible, IFN-mediated anti-ASV activity in mammalian cells does not require Daxx. Together with our previous demonstration that Daxx is essential for anti-retroviral host defense in the absence of IFNs [2,3], our current observations support a model for Daxx function in which Daxx protects against ASV (by epigenetic repression of ASV proviral gene expression) prior to induction of IFNs. Once induced, type I IFNs can establish an anti-retroviral state in which Daxx is not essential.

A Daxx ortholog has been identified in *Drosophila* and other insects [12], predating by ~150 million years the emergence of IFN-α/β genes, which can be traced back ~250 million years to the time when reptiles and birds diverged from each other [13,14]. It is thus possible that Daxx represents an ancient, metazoan anti-retroviral protein the function of which remains essential in the absence of IFNs, but which has since been rendered redundant by the relatively-recent emergence of the type I IFN system in higher vertebrates.

Alternatively, our finding that IFN-mediated protection against ASV is Daxx-independent may be explained simply by activation of mechanistically distinct, but functionally redundant ISGs. Indeed, dependence on a single ISG may be detrimental to the host in the face of a virus infection, as viruses are capable of rapid evolution and consequent subversion of antiviral host proteins. Several viruses are known to target Daxx. For example, the human cytomegalovirus (HCMV) virion tegument protein pp71, as well as the adenovirus E1B-55 K protein have been shown to induce degradation of Daxx via the proteasome [15]. Redundant antiviral mechanisms ensure that multiple host defense strategies are in place, should any one, e.g. Daxx, be compromised by virus infection. We speculate that in mammalian cells, IFNs target multiple early steps in the ASV life cycle upstream of where Daxx is proposed to act, including entry, capsid disassembly, uncoating, nuclear entry/reverse transcription, and integration. As IFNs are capable of anti-ASV activity even in the absence of Daxx, epigenetic repression of proviral DNA by Daxx likely represents only one of the many diverse pathways, including those activated by APOBEC3G, TRIM5, TRIM22, and MXB, which are deployed by IFNs to restrict early steps of retroviral replication [16-18]. For example, APOBEC3G triggers damaging hypermutation of retroviral cDNA following reverse transcription, TRIM5 blocks HIV-1 by inhibiting viral cDNA synthesis, and MXB has been reported to inhibit HIV-1 DNA integration [16-18]. Induction of these or similar restriction factors may account for IFN-mediated protection against ASV in the absence of Daxx.

## Competing interests

The authors declare that they have no competing interest.

## Authors’ contributions

KAH performed most of the experiments and wrote the manuscript. NS and SN generated virus stocks and assisted with experiments. RK, AMS, and SB conceived this study and edited the manuscript. All authors read and approved the final manuscript.
